# The impact of four years of semiannual treatments with albendazole alone on lymphatic filariasis and soil-transmitted helminth infections: A community-based study in the Democratic Republic of the Congo

**DOI:** 10.1371/journal.pntd.0008322

**Published:** 2020-06-23

**Authors:** Sébastien D. S. Pion, Cédric B. Chesnais, Naomi P. Awaca-Uvon, Johnny Vlaminck, Anlimou Abdou, Billy Kunyu-Shako, Godefroy Kuyangisa Simuna, Jean-Paul Tambwe, Gary J. Weil, Michel Boussinesq

**Affiliations:** 1 French National Research Institute for Sustainable Development, Montpellier, France; 2 Ministry of health, Kinshasa, Democratic Republic of the Congo; 3 Ghent University, Merelbeke, Belgium; 4 National Institute of Biomedical Research, Kinshasa, Democratic Republic of the Congo; 5 Washington University School of Medicine, St. Louis, Missouri, United States of America; George Washington University School of Medicine and Health Sciences, UNITED STATES

## Abstract

**Background:**

The World Health Organization now recommends semiannual mass drug administration (MDA) of albendazole with integrated vector management as an option for eliminating lymphatic filariasis (LF) in areas of loiasis-endemic countries where it may not be safe to use diethylcarbamazine or ivermectin in MDA programs. However, the published evidence base to support this policy is thin, and uptake by national programs has been slow.

**Methodology/Principal findings:**

We conducted a community trial to assess the impact of semiannual MDA on lymphatic filariasis and soil-transmitted helminth infections (STH) in two villages in the Bandundu province of the Democratic Republic of the Congo with moderately high prevalences for LF and hookworm infections. MDA with albendazole was provided every six months from June 2014 to December 2017 with treatment coverages of the eligible population (all ≥ 2 year of age) that ranged between 56% and 88%. No adverse effects were reported during the trial. Evaluation at 48 months, (i.e. 6 months after the 8^th^ round of MDA), showed that *W*. *bancrofti* microfilaremia (Mf) prevalence in the study communities had decreased between 2014 to 2018 from 12% to 0.9% (p<0.001). The prevalence of *W*. *bancrofti* antigenemia was also significantly reduced from 31.6% to 8.5% (p<0.001). MDA with albendazole also reduced hookworm, *Ascaris lumbricoides* and *Trichuris trichiura* infection prevalences in the community from 58.6% to 21.2% (p<0.001), from 14.0% to 1.6% and 4.1% to 2.9%, respectively. Hookworm and *Ascaris* infection intensities were reduced by 93% (p = 0.02) and 57% (p = 0.03), respectively. In contrast, *Trichuris* infection intensity was not significantly reduced by MDA (p = 0.61) over this time period.

**Conclusion/Significance:**

These results provide strong evidence that semiannual MDA with albendazole alone is a safe and effective strategy for LF elimination in Central Africa. Community MDA also had a major impact on STH infections.

## Author summary

In low-income rural settings of Africa, populations are commonly affected by multiple parasitic diseases. Some of these diseases have been targeted for elimination through dedicated national or international programs. In 2012, the World Health Organization (WHO) defined a specific strategy to eliminate lymphatic filariasis, the disease responsible for elephantiasis, in central Africa. This strategy consists in treating the whole population living in endemic areas every six months with a single donated drug—albendazole. Together with the use of the night bed nets distributed as part of malaria programs, it was expected that this strategy could interrupt the transmission and eliminate lymphatic filariasis locally within period of 4 to 7 years. Here, we evaluated this strategy in two endemic communities near Bandundu in the Democratic Republic of the Congo. We also assessed the impact of the semiannual community treatments on three species of gastrointestinal parasitic worms. Our results suggest that semiannual MDA with albendazole is effective for LF elimination in Central Africa, but they also indicate that drug-based only intervention is not enough to eliminate gastrointestinal worm infections in areas with high transmission.

## Introduction

Lymphatic filariasis (LF) in Africa is caused by *Wuchereria bancrofti* and is transmitted mainly by *Anopheles* or *Culex* mosquitoes. In 2000, the World Health Organization (WHO) launched an elimination program for LF that was based on an innovative rapid mapping procedure to identify endemic areas, and annual mass drug administration (MDA) of albendazole (ALB, 400 mg) plus ivermectin (150–200 μg/kg) in countries where onchocerciasis is coendemic with LF, and with ALB plus diethylcarbamazine (DEC) in countries with no onchocerciasis. More than 7 billion treatments were delivered to populations in need between 2000 and 2017, and this has had a substantial impact on infection prevalence and intensity of transmission [1]. However, the presence of *Loa loa* in Central Africa prevents the widespread use of ivermectin for LF elimination in areas that are not already receiving ivermectin for onchocerciasis control, because ivermectin sometimes causes serious adverse events that can include coma and death in individuals with high *L*. *loa* microfilaremia [2]. This situation represents a major challenge for LF elimination in Africa.

WHO proposed a provisional strategy for controlling LF in areas with coendemic loiasis but where onchocerciasis is absent in 2012. This consists of MDA using ALB (preferably with semiannual delivery) together with integrated vector management [3]. It has been estimated that 1.4 million people live in areas potentially targetable with this treatment scheme. [4] So far, this strategy has been supported by results from a single published study that was conducted in an area in the Republic of the Congo where baseline circulating filarial antigenemia (CFA) and microfilaremia (Mf) prevalences were moderate (17.3% and 5.3%, respectively) [5]. In June 2014, we started a parallel trial in an area of the Democratic Republic of the Congo with significantly higher baseline infection prevalences. This paper reports the impact of eight rounds of MDA with ALB on LF and on STH in this high endemicity setting.

## Methods

### Study design

The objective of this study was to assess the impact of semiannual community treatments with ALB on LF and STH over a 4-year period. The study was conducted in Mbunkimi (3°31′41″S, 17°37′44″E) and Misay (3°30′44″S, 17°37′29″E), two contiguous villages located in a savanna area of the Kwilu province, in the Democratic Republic of the Congo. Due to their proximity and similarity, the two villages were considered to be one community for this study. An exhaustive census of the population was conducted in April 2014, approximately one month before the start of the trial. The census was updated prior to each round of MDA to enable precise calculations of treatment coverage (see below). Baseline and post-treatment parasitological assessments for LF and STH were conducted between mid-June and mid-July for the years 2014, 2015, 2016, 2017 and 2018. MDA using ALB was conducted every year just after the parasitological examinations, and also in late December 2014, 2015, 2016 and 2017. Therefore, each parasitological assessment occurred 6 months after the previous MDA. Annual parasitological assessments were performed for those ≥ 5 years of age. During each round of MDA, ALB was also offered to all children aged 2–4 years of age, but they were not targeted for parasitological assessment.

### Detection of *W*. *bancrofti* infections

*W*. *bancrofti* infections were detected by antigen testing with mf testing reserved for those with positive antigen tests. CFA was detected with the Filariasis Test Strip (FTS) (Alere-Scarborough, Scarborough, ME) according to the manufacturer’s instructions. The results were scored semi-quantitatively as previously described [6]. All individuals with a positive FTS (score ≥ 1) were invited to return for blood sampling between 10:00 PM and 1:00 AM for assessment of *W*. *bancrofti* mf. Fingers were cleaned with an alcohol wipe and pricked with a 1.5 X 2.0 mm BD Microtainer contact activated lancet (Becton Dickenson, Franklin Lakes, NJ). Blood was collected with a capillary tube, and two blood smears (volume 70 μL each) were prepared for each subject. On the next day, the blood smears were dehemoglobinized, stained with Giemsa, and examined by two experienced microscopists. Each microscopist read one of the two slides from each subject. Differences of >10% in Mf counts were resolved by re-examination of both slides. The Mf count for individual subjects was defined as the arithmetic mean of the counts from the two slides and reported as mf per milliliter of blood (mf/mL).

### Detection of STH infections

Every day, about 10 households listed in the census register were visited, and all occupants aged 5 years or older were asked to report for testing and treatment on the next day. They were also given a 50 mL plastic stool container and asked to collect a sample of their stool the following morning. The stools specimens were collected every day at around 9 AM, stored in cooling boxes with ice packs and shipped within 6 hours to the laboratory where they were placed in a refrigerator at 6°C. From each sample, two thick smears were prepared within 24 h of collection according to the modified Kato-Katz method [7]. Slides were examined by microscopy at a 40x magnification within 1 hour after preparation, and results were reported as eggs per gram of stool (epg).

### Drug distribution, treatment coverage and bednet usage

Individuals who had a negative CFA result were immediately treated with ALB (400 mg) which was taken on the spot under the direct observation of investigators. Those with positive CFA results received the drug just after collection of night blood for mf testing. Finally, all inhabitants who had not participated in the parasitological survey or who missed the night blood testing (due to absence or refusal) were later visited at home and offered ALB treatment. All drugs were distributed under the supervision of a local healthcare worker who was also in charge of the population census. For treatment of young children, ALB tablets were crushed with a spoon and mixed with water. Although WHO considers that pregnant women can be treated with ALB during the second or third trimester, we decided to exclude all pregnant women from MDA. ALB was offered to them by the local healthcare worker after they gave birth. The treatment coverage was calculated as the number of individuals who received the drug divided by the population aged ≥ 2 years old as recorded in the census prior to the MDA. For each individual, we computed the total number of ALB doses received during the study according to the information reported in treatment books. Bed net usage was assessed with the question “Did you sleep under a bed net last night?”

### Statistical analysis

The cumulative number of treatments received by the participants who were present in June 2018 was analyzed as a function of age and gender using an analysis of variance followed by a Tukey HSD post hoc test. Infection prevalence in the population between the baseline examination round and year 4 were compared independently for each parasite with the Chi-squared test. Infection intensities for each *W*. *bancrofti* microfilaremia (mf/mL) and for STH infection (eggs per gram, epg) were compared using the Mann-Whitney test. A longitudinal analysis of prevalence and infection intensities was conducted in a subgroup of individuals who were examined both at baseline and at year 4 using McNemar and Wilcoxon signed ranks tests, respectively. Mean infection intensity measures for *W*. *bancrofti* (mf/mL) and STH (epg), are reported as geometric means of positive counts (GM) or arithmetic means (AM).

The prevalence of *W*. *bancrofti* microfilaria carriers in the community was calculated assuming that persons with negative CFA results had mf counts of zero, and the community microfilarial load was calculated as the Williams geometric mean (i.e. the geometric mean of (Mf counts+1) minus 1). For STH infections, we considered classes of infection intensity in accordance with the WHO guidelines [8].

### Ethical clearance

This study was approved by the Health Ethics Committee (Comité National d'Ethique de la Santé, CNES) of the Democratic Republic of the Congo and conducted with personnel of the Ministry of Public Health of the country. The purpose of the study was verbally presented during meetings with village leaders and then to all study participants in both French and Lingala (national official language). A document written in French and in Lingala detailing the study purpose and activities was also given to each individual. Adult participants signed an informed consent form. Participants younger than 18 years of age were enrolled only if they expressed verbal assent to participate in the study and if at least one parent signed a parental consent form.

## Results

### Study participants and treatment coverage

In June 2014, Mbunkimi and Misay had a total population of 1290 inhabitants, among which 1033 were ≥ 5 years of age. The population size, number of participants who underwent CFA testing, night blood smears sampling and who provided stools are presented in Table 1. There were no exclusion criteria regarding CFA testing. Therefore all consenting residents of the study communities ages > 5 years were tested for CFA regardless of whether they were eligible for treatment.

**Table 1:** CFA, circulating filarial antigenemia by the Filariasis Test Strip (FTS).

	June 2014	Dec. 2014	June 2015	Dec. 2015	June 2016	Dec. 2016	June 2017	Dec. 2017	June 2018
Total Population	1266	1276	1316	1336	1335	1321	1324	1296	1278
Pop ≥ 2 years	1177	1197	1194	1241	1233	1260	1299	1273	1275
Treated (% of Pop ≥ 2 years)	924 (78.5)	902 (75.4)	846 (70.9)	1046 (84.3)	878 (71.2)	1106 (87.8)	815 (62.7)	1077 (84.6)	714 (56.0)
No. participants for LF assessment	820	-	795	-	766	-	701	-	531
Age, years*	20 (10–35)	-	18 (10–33)	-	18 (10–34)	-	16 (9–33)	-	19 (11–35)
Sex-ratio (M/F)*	0.88	-	0.96	-	0.82	-	1.12	-	1.29
No. CFA+(% of examined)	259 (31.6)	-	228 (28.7)	-	158 (20.6)	-	107 (15.3)	-	45 (8.5)
No. Night blood smears	249	-	202	-	138	-	95	-	43
No. Night blood smears with mf (%)^1^	97 (39%)	-	62 (30.7%)	-	29 (21%)	-	13 (13.7%)	-	5 (11.6%)
No. stool samples	413	-	214	-	308	-	251	-	184
Age, years^§^	19 (10–36)	-	16.5 (8–39)	-	15 (8–35)	-	10 (8–15)	-	10 (8–12)
Sex ratio^§^	0.75	-	1.00	-	0.96	-	1.21	-	1.79
Bednet usage (verbal report)*	61.1%	-	50.6%	-	98.3%	-	63.6%	-	46.1%

Mf, microfilaremia

^1^ percentage of blood smears with microfilariae in individuals with positive CFA test results

Data are n, n (%), or median (Inter Quartile Range), unless otherwise specified

* In patients tested with FTS.

^§^In patients tested for soil-transmitted helminths

The treatment coverage ranged between 56% and 87.8% during the 5-year period (Table 1). No adverse events were reported after the 8308 ALB treatments distributed throughout the study. Among the 531 individuals who were examined in June 2018, the median number of ALB doses received between June 2014 and December 2017 was 7 (Interquartile range IQR: 5–8). The total number of albendazole doses was not dependent on gender (p-value = 0.4033) but varied across age-groups (p-value = 0.0021), with the lowest value recorded in the 20–29 year-old group (mean = 3.8) and the highest values in the 5–9 (mean = 5.35) and 10–14 (mean = 5.55) groups.

### Impact of eight semiannual community ALB treatments on lymphatic filariasis in the community

**Fig 1:**
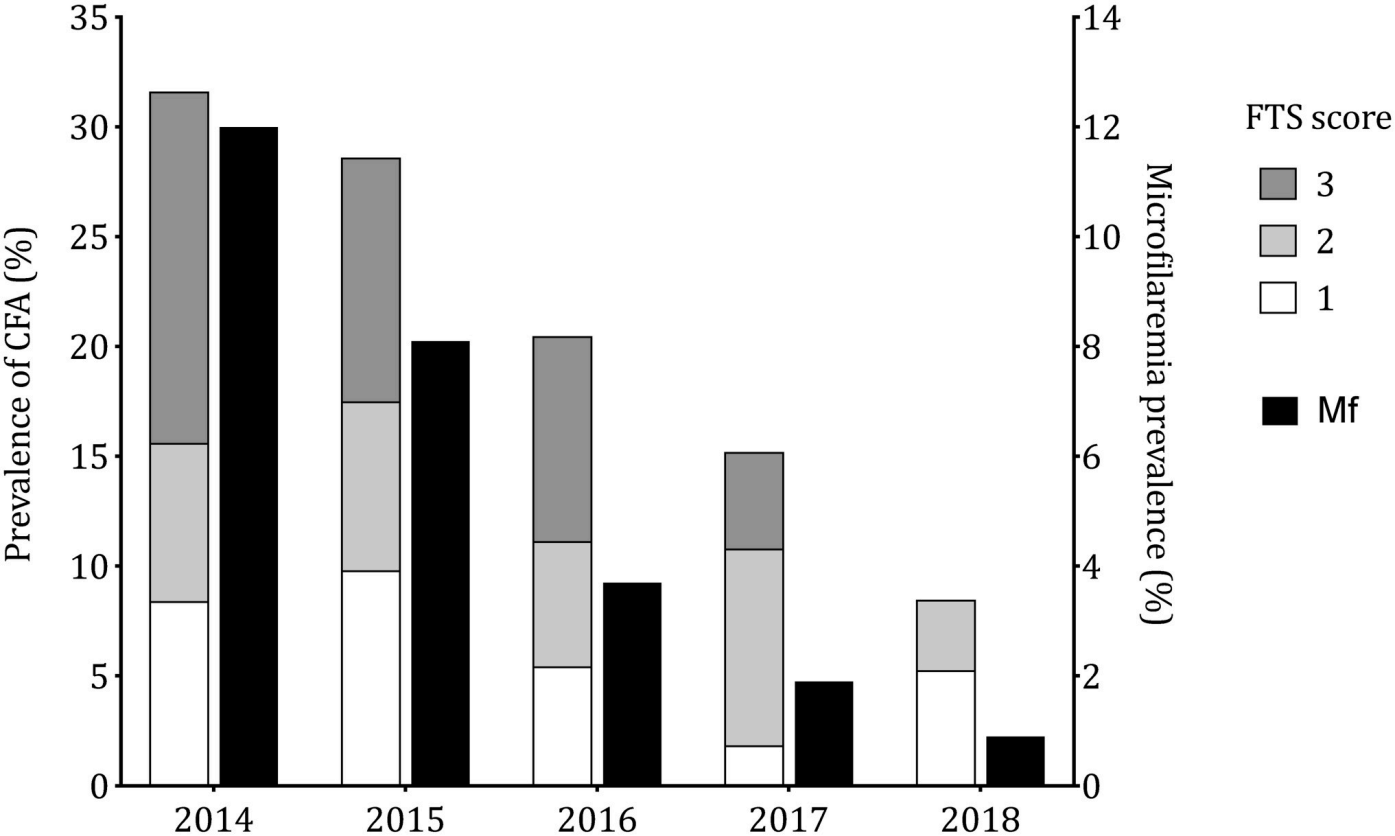
CFA and microfilaremia rates during the 4-year period.

**Fig 2:**
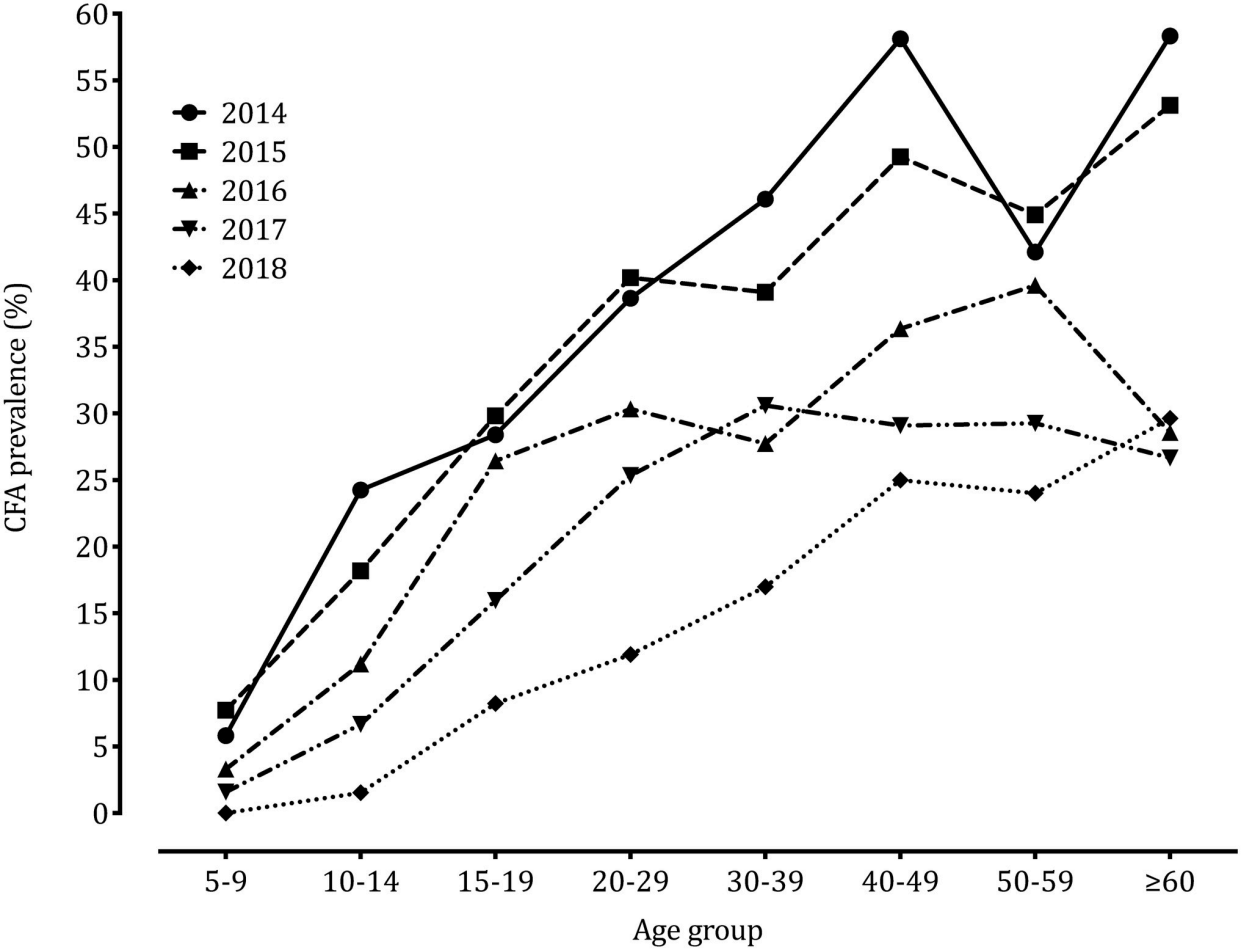
Evolution of age profiles of CFA prevalence from 2014 to 2018.

The CFA prevalence in the community decreased from 31.6% (95% confidence interval, CI: 28.5–34.9) in 2014 to 8.5% (95% CI: 6.4–11.2) in 2018 (p-value<0.001, see Fig 1 for interim CFA rates). The most important drop in CFA prevalence was observed in the 40–49 year-old age group: CFA dropped from 58.1% (95% CI: 46.4–68.9) in 2014 to 25% (95% CI: 13.2–42.1) in 2018 (Fig 2). Of note, in 2018, there were no CFA carriers in the youngest age-group (Fig 2). In 2018, contrary to observations from the previous years, most positive FTS had scores of 1 (low intensity) and no FTS had scores of 3 (high intensity) (Fig 1). Assuming that all individuals with a negative CFA test do not harbor microfilariae, the estimated Mf prevalence in the community decreased from 12% (95% CI: 9.9–14.4) in 2014 to 0.9% (95% CI: 0.4–2.3) in 2018 (p-value<0.001). Mf density calculated as the geometric mean of positive counts decreased from 171 mf/mL in 2014 to 70.6 mf/mL in 2018 (p-value = 0.0004, Fig 1) and the community Mf load decreased from 0.9 mf/mL in 2014 to 0.06 mf/mL in 2018.

### Impact of eight semiannual community ALB treatments on lymphatic filariasis in a sub-group of individuals examined both in 2014 and 2018

Effect of ALB on *W*. *bancrofti* was assessed in a subset of 320 individual who were tested in 2014 and in 2018 to assess impact of annual MDA on LF infection intensity. Ninety-six (30%) had positive CFA tests at baseline; 59 of 96 persons in this group (61.5%) had negative antigen test results in 2018. 35 (36.5%) with persistent filarial antigenemia had lower intensity scores. One (1%) participant had an increased score (from 1 to 2) and one (1%) had the same score of 1 in 2014 and 2018 (Table 2). Of note, none of 224 CFA negative individuals at baseline had positive CFA tests in 2018. Mf prevalence and geometric mean of positive mf counts in the group of 320 individuals with a 4-year follow-up decreased from 11.6% to 1.3% (p-value<0.0001) and from 291.2 mf/mL (in 37 positive individuals) to 125.1 mf/mL (in 4 positive individuals) (p-value = 0.0003), respectively.

**Table 2:** Evolution of circulating filarial antigenemia (FTS) scores in 320 individuals tested in 2014 and in 2018.

		FTS score in June 2018				
		0	1	2	3	Total
FTS score in June 2014	0	224	0	0	0	224
	1	23	1	1	0	25
	2	20	4	0	0	24
	3	16	18	13	0	47
	Total	283	23	14	0	320

### Impact of eight semiannual community ALB treatments on soil transmitted helminth infections

**Fig 3:**
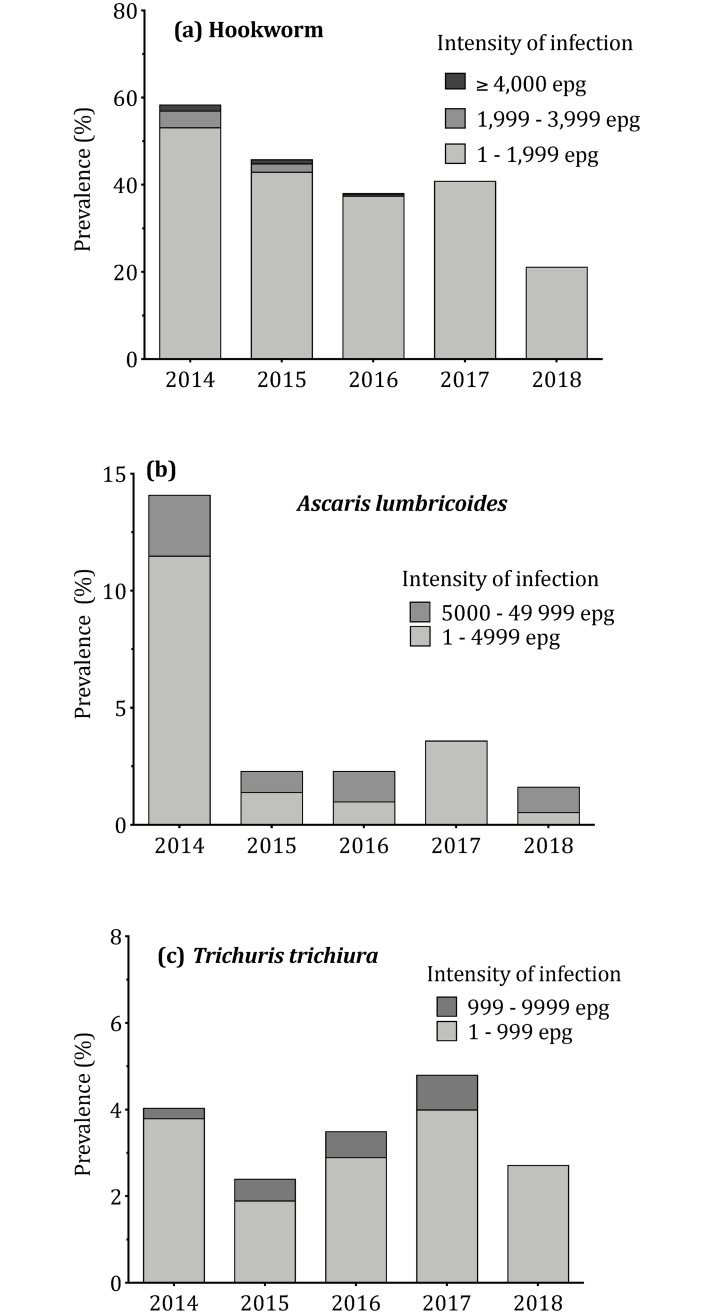
Impact of semiannual treatments with albendazole on soil-transmitted helminth infections *epg: eggs per gram.

The prevalence of hookworm infection in the community decreased progressively from 58.6% in 2014 to 21.2% in 2018 (p-value<0.001, Table 3). The arithmetic mean egg count for hookworm decreased from 456 epg in 2014 to 31 epg in 2018 (p-value<0.0001). In 2018, all remaining infected individuals were in the lowest WHO category for infection intensity (< 2000 epg) (Fig 3a). The prevalence of *A*. *lumbricoides* infection in the community decreased from 14% in 2014 to 1.6% in 2018 (p-value<0.0001). The arithmetic mean of *A*. *lumbricoides* egg counts decreased from 578 epg in 2014 to 173 epg in 2015 to 248 epg in 2018 (p-value = 0.0858). None of the participants had more than 50,000 *A*. *lumbricoides* epg (high intensity per WHO’s classification [8]) during the study (Fig 3b). The community prevalence of *T*. *trichiura* infection was 4.1% in 2014 and 2.9% in 2018 (p-value = 0.2499). Arithmetic mean egg counts were 8 epg in 2014, 10 epg in 2015, 31 epg in 2016, 28 in 2017 and 3 epg in 2018. All infections detected in 2018 had egg counts lower than 1000 epg (Fig 3c).

**Table 3:** Effect of semiannual mass administration of albendazole on soil-transmitted helminth infections.

	2014 (N = 413)	2015 (N = 214)	2016 (N = 308)	2017 (N = 252)	2018 (N = 184)	% reduction 2014–2018	p-value 2014–2018
Rounds of mass drug administration before assessment	0	2	4	6	8		
Hookworm							
Number of positive samples	242	98	118	103	39		
Prevalence (95% CI)	58.6 (53.7–63.3)	45.8 (39.2–52.5)	38.3 (33.0–43.9)	40.9 (34.9–47.1)	21.2 (15.9–27.7)	63.8%	< 0.0001
Arithmetic mean eggs per g (95% CI)	456.1 (319.2–593.0)	251.0 (154.01–348.1)	111 (66.7–155.3)	66.9 (45.6–88.2)	31.0 (14.8–47.1)	93.2%	< 0.0001
Geometric mean eggs per g (95% CI)	210.9 (171.6–259.1)	196.5 (45.0–266.2)	112.4 (88.0–143.6)	80.3 (63.4–101.7)	70.6 (48.3–103.3)		
*Ascaris lumbricoides*							
Number of positive samples	58	5	7	9	3		
Prevalence (95% CI)	14.0 (11.0–17.8)	2.3 (1.0–5.5)	2.3 (1.1–4.7)	3.6 (1.9–6.7)	1.6 (0.5–5.0)	88.6%	< 0.0001
Arithmetic mean eggs per g (95% CI)	578.4 (305.1–851.6)	173.0 (-84.5–430.5)	176.5 (1.5–351.5)	30.3 (-0.1–60.7)	247.6 (-114.6–609.7)	57.2%	0.0858
Geometric mean eggs per g (95% CI)	972.6 (569.6–1660.7)	524.9 (5.7–47934.8)	2475.3 (297.2–20616.6)	336.0 (86.8–1299.8)	10438.1 (693.7–157058.3)		
*Trichuris trichiura*							
Number of positive samples	17	5	11	12	5		
Prevalence (95% CI)	4.1 (2.6–6.5)	2.3 (1.0–5.5)	3.6 (1.9–6.3)	4.8 (2.7–8.2)	2.7 (1.1–6.4)	34.0%	0.2011
Arithmetic mean eggs per g (95% CI)	8.0 (-0.8–17.0)	10.5 (-4.9–25.9)	31.3 (-11.7–74.3)	28.4 (-12.9–69.6)	3.4 (0.2–6.6)	57.5%	0.2499
Geometric mean eggs per g (95% CI)	59.7 (27.3–130.7)	163.9 (17.8–508.8)	72.0 (14.8–350.8)	83.4 (24.7–281.5)	112.7 (57.6–220.6)		

## Discussion

This study represents the longest and largest trial to date of semiannual community treatment with ALB alone for LF elimination. Our results show that semiannual ALB was effective for reducing LF prevalence in an area with moderately high endemicity in the Democratic Republic of the Congo. MDA also greatly reduced STH prevalences and intensity in the study area. Eight semiannual rounds of MDA with ALB decreased the CFA prevalence in the study communities by 73% from 31.6% to 8.5% and decreased the Mf prevalence by 92.5% from 12% to 0.9%. Of note, no child aged less than 10 years of age had a positive CFA test result in 2018, and no person who was CFA-negative in 2014 was positive in 2018. Although we have no data on CFA incidence rates prior to MDA, these results suggest that MDA dramatically reduced LF incidence in the study area. Results from this study were similar to those obtained in a prior community study of semiannual ALB MDA that was conducted in the Republic of the Congo, about 400 km from the current study site [5]. However, baseline LF prevalences for CFA and mf in that area were only about half as high as those in the current study. Taken together, these studies strongly support the provisional strategy for elimination of LF in Central Africa that was first issued by the WHO in 2012 and reaffirmed in 2017 [9]. They also suggest that LF transmission might be interrupted within a 5-year time frame in LF endemic areas in Central Africa if programs can implement semiannual MDA with ALB with high compliance.

The absence of SAEs in this study was welcome but not unexpected, given the known safety record of ALB in LF elimination and STH control programs [10,11]. Although semiannual MDA with ALB reduces mf prevalence more slowly than ivermectin plus albendazole during the first year of intervention [12], it may be a safer regimen for LF elimination than IVM/ALB, and that is particularly likely to be true in areas of Central Africa where LF is coendemic with loiasis. However, a potential downside of MDA with ALB alone is that it may select for drug resistance in nematode parasites if it is continued for many years [13].

A graph of treatment coverage over time revealed a saw-tooth pattern, with coverage oscillating between 65% and 85% with lower values when the treatments were associated with the annual parasitological assessments. The last round saw a significant drop in participation (56%), possibly reflecting program fatigue on the part of some members of the community. If community treatments have to be maintained for longer periods, one of the main challenges will be to find ways to reinforce messages to improve and maintain compliance with MDA.

It is not surprising that community MDA with ALB had dramatic effects on hookworm and ascariasis infections in the study communities [14,15]. It is also not surprising that semiannual ALB had only modest effects on *Trichuris* prevalence, because *Trichuris* is known to be less susceptible to ALB than the other STH [14,15]. It is interesting to compare STH results from this study to those reported from Seke Pembe in the Republic of the Congo [5]. Baseline STH prevalences in this study differed significantly from those in Seke-Pembe: Hookworm was the preponderant STH species in the present study (initial prevalence: 58.6%), followed by *A*. *lumbricoides* (14%) and *T*. *trichiura* (4.1%), whereas corresponding baseline prevalences in Seke-Pembe were 6.5%, 56.5% and 78.6%, respectively. In the latter site, two rounds of albendazole led to the total disappearance of hookworm infection in the community. In the present study site, the prevalence of hookworm infection was still slightly above 20% after 8 rounds of community treatments. Reinfection with hookworm was certainly reduced, but it is still occurring in Mbunkimi and Misay.

The prevalence of *A*. *lumbricoides* infection seemed to level off at a low value (2–3%) after a dramatic reduction between the baseline year and year-1. Albendazole had little effect on *T*. *trichiura* infection prevalence which was already low at baseline. Additional studies would be needed to assess whether the small reservoirs of *A*. *lumbricoides* and *T*. *trichiura* are aggregated within related individuals or locations in the study area. If that is the case, a different treatment schedule or regimen might be employed to treat infected individuals and their family members or to focus treatment for individuals who live in specific sectors. However, based on our results, elimination of STH in the community is unlikely to be achieved using semiannual albendazole alone.

This study has some limitations. First of all, for both logistical and ethical reasons, we chose not to include a control community to monitor the level of *W*. *bancrofti* and STH infections without intervention. Nonetheless, during the 4-year study, there were no significant changes in the study communities, such as improvement of sanitary conditions or insecticide campaigns, that may have impacted the transmission of LF and STH. Besides this, bednets that were present in the households were not distributed as part of our trial. They were already present in the village at the outset of the trial. We did not strictly assess their condition but fortuitous observations showed very degraded nets. In addition, although a prior study showed that long-lasting insecticidal nets used during malaria programs can interrupt LF transmission, that study also reported that Mf prevalence was not significantly affected during the 3-year study [16]. Therefore, we firmly believe that the fast decrease in both CFA and Mf prevalence is due to the effect of repeated treatments with albendazole.

Finally, one factor to consider regarding STH results in 2018 is that the stools submitted for examination were almost entirely provided by children. Therefore, according to what is known about the age-profiles of the different STH species, STH prevalences in the community may have been overestimated for *Ascaris* and *Trichuris*, but underestimated for hookworm. In addition, persons in the study area who were noncompliant with MDA and not tested for STH may also constitute an unmeasured reservoir of STH infection.

To conclude, our findings indicate that 4 years of semiannual community treatments with albendazole alone with high treatment coverage decreased the Mf prevalence in the community to less than 1%. In programmatic terms, this sentinel site has passed pre-TAS. However, this is not sufficient to trigger a transmission assessment survey (TAS). TAS are conducted at the scale of evaluation units. Although our study started in 2014, semiannual MDA with albendazole started later in the rest of health area. Therefore, we would deferring TAS in this area until all communities in the implementation unit have completed 4 years of semiannual MDA with albendazole.

## Supporting information

S1 Checklist STROBE checklist. (DOC)

S1 Dataset Individual data used to produce all tables and graphs included in the article. (XLSX)
